# Trends in the incidence of oral cancer in Saudi Arabia from 1994 to 2015

**DOI:** 10.1186/s12957-020-01989-3

**Published:** 2020-08-20

**Authors:** Bandar M. Alshehri

**Affiliations:** Department of Clinical Laboratory, Faculty of applied Medical Sciences, Najran University, P.O. Box: 1988, Najran, Kingdom of Saudi Arabia

**Keywords:** Cancer epidemiology, Cancer prevention and control, Oral neoplasm

## Abstract

**Background:**

Oral cancer is one of the most common non-communicable diseases worldwide. This paper presents an evaluation of the trends and geographical distributions of oral cancers in the Saudi Arabian population.

**Methods:**

Data from Saudi Cancer Registry reports were used in this analysis, which assessed the period between 1994 and 2015. All cancer cases are recorded in these reports, as well as the age, gender, region and histological cancer sites for each patient. Age-standardised and age-specific incidence rates were calculated in these reports. For the purposes of this paper, only cancers of the lips, tongue and mouth were considered oral cancers.

**Results:**

Between 1994 and 2015, the Saudi Cancer Registry identified 172,424 cancer cases in total. Of these, 3184 were oral cancer. The mean age-standardised rate of oral cancer for the study period was 2.9 per 100,000 people; for females, it was 1.5, and for males, it was 1.4. The incidence of oral cancer varied by region, with Jazan displaying the highest age-standardised rate and Hail displaying the lowest. A positive correlation was observed between oral cancer incidence and age.

**Conclusion:**

The overall trend of the age-standardised rate for both sexes remained constant from 1994 to 2015. However, the oral cancer incidence in Saudi Arabia varies by region. Studying this variation in more detail will help to guide awareness programmes in the regions that are most in need.

## Background

Cancer is an intractable global health problem and the leading cause of death in the developed world; in the developing world, it is the second-leading cause [1]. In 2018, the most recent year for which information from the International Agency for Research on Cancer (IARC) is available, approximately 18.1 million new cancer cases were diagnosed, and 9.5 million people died from cancer worldwide [2]. In this same year, 354,864 new cases of lip and oral cavity cancers were reported, representing 2% of all cancer cases.

A review of the global prevalence of oral cancer reveals a wide variation in distribution among countries [3]. Two-thirds of the estimated incidence of oral cancer occurred in developing countries, with up to 25% of all new oral cancer cases in Sri Lanka, India, Pakistan and Bangladesh [3]. Conversely, in France, which has the highest rate of oral cancer incidence in the European Union, only 15,500 oral cancer cases were reported in 2004, representing just 5.5% of all cancer cases [3]. In the USA, the American Cancer Society estimated that in 2019, approximately 53,000 people were diagnosed with oral cavity or oropharyngeal cancer, and 10,860 will die of these cancers [4]. In Arab countries, the prevalence of oral cancer is concentrated between western and southeast Asia [5]. While this type of cancer is relatively uncommon across Arab gulf countries, Saudi Arabia and Yemen are notable exceptions [5]. No studies have been published discussing the epidemiological parameters and geographic distribution of oral cancer cases or any of its subtypes in the Saudi Arabian population. Therefore, this study analysed and discussed oral cancer trends in the Saudi population by using the most recent data available.

According to the International Classification of Diseases, 10th revision (ICD-10), oral cancer is classified into six sites: mucosal lip (ICD-10: C00), tongue (ICD-10: C02), gum (ICD-10: C03), mouth floor (ICD-10: C04), palate, (ICD-10: C05) and mouth (ICD-10: C06). However, examining trends in oral cancer incidence rates that include all oral sites can be misleading. The data analysed in this study only include cancers of the lip, tongue and mouth (ICD-10:C00–C06), which form the majority of oral cancers; moreover, they have several risk factors in common and share a similar biology [6]. Thus, those accounting for a minority of oral cancer cases were excluded.

## Materials and methods

### Data

This retrospective descriptive epidemiological study analysed oral cancer cases in a Saudi population that had been diagnosed from January 1994 through December 2015. The study used a method of analysis similar to that used by Alshehri et al. [7]. Their analyses incorporated male and female data on lip, tongue and mouth (ICD-10:C00–C06) cancer cases to evaluate disease patterns in the Saudi population. Data for the present study were obtained from the Saudi Cancer Registry (SCR), a population-based registry established in 1994 by the Ministry of Health in Saudi Arabia. This data can only be obtained from the reports published by the SCR.

Since 1994, the SCR has been publishing reports on cancer in Saudi Arabia with the primary objective of defining population-based cancer incidences. The present study was conducted using these reports to derive a descriptive epidemiology of oral cancer in Saudi Arabia. In SCR reports, age-standardised (ASR) and age-specific (AIR) incidence rates were calculated, with a focus on gender-specific and regional differences.

The analysis included cases recorded in the SCR files from January 1994 to December 2015, totalling 172,424 cancer cases overall, approximately 3184 of which were oral cancer.

### Data analysis

The GraphPad Prism6 software was used to analyse the data. Descriptive analyses of epidemiological data were conducted by calculating the mean of the percentages and ASR stratified by age, sex, region and year of diagnosis. The ASR was calculated in the SCR reports by adjusting all Saudi regions’ populations mathematically to have the same age structure. On the other hand, the AIR was calculated by summation of the number of cancer cases occurring during the year in a region’s population among specific age and sex groups divided by the midyear population of these age and sex groups.

Using these two standardised rates is important because age is a basic element of the risk of developing cancer globally [8]. Using summary measure tools, such as the ASR and AIR, which represent the schedule of age-specific rates in different regions and across time, will give us a more representative picture of the characteristics in question and enable comparisons of cancer incidences between several populations of Saudi regions that differ with respect to age.

## Results

### Increase in the number of oral cancer cases

The total number of cancer cases identified by the SCR from 1994 to 2015 was 172,424, with 83,185 (48.2%) males and 89,239 (51.7%) females. Of this total, 3184 cases (1.8%) were oral cancer. The number of registered oral cancer cases increased gradually from 109 (63/46 M/F) in 1994 to a peak of 211 (121/90 M/F) in 2014; however, only 175 cases were reported in 2015 (96/79 M/F) (Table 1).

**Table 1:** Number of oral cancer cases in Saudi Arabia for the period from 1994 to 2015

Year	Number of male cases	Number of female cases	Total
**1994**	**63**	**46**	109
**1995**	**39**	**46**	85
**1996**	**49**	**57**	106
**1997**	**58**	**56**	114
**1998**	**45**	**64**	109
**1999**	**55**	**77**	132
**2000**	**57**	**53**	110
**2001**	**40**	**50**	90
**2002**	**51**	**64**	115
**2003**	**56**	**66**	122
**2004**	**74**	**58**	132
**2005**	**78**	**71**	149
**2006**	**73**	**64**	137
**2007**	**88**	**96**	184
**2008**	**78**	**80**	158
**2009**	**87**	**82**	169
**2010**	**106**	**96**	202
**2011**	**93**	**105**	198
**2012**	**83**	**88**	171
**2013**	**102**	**104**	206
**2014**	**121**	**90**	211
**2015**	**96**	**79**	175

**Fig. 1:**
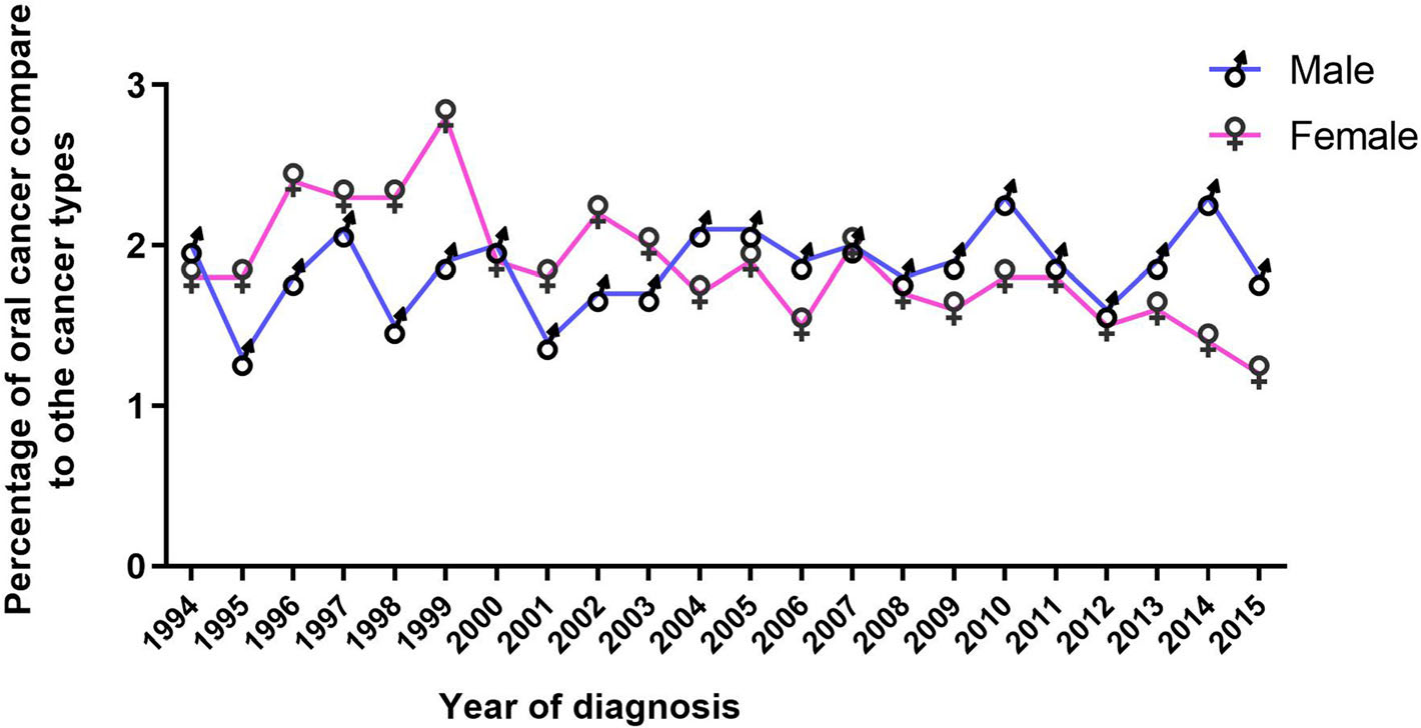
Consistency in percentage curves for oral cancer out of all cancer types from 1994 to 2015. The percentage curve for oral cancer out of all cancer types for males and females are correlated with overall increases and decreases over the period from 1994 to 2015, with the exception of years 1998 and 1999

The percentage of cases representing oral cancers was 1.8% for females and 2.0% for males (Fig. 1) in 1994. These percentages decreased to 1.6% for females and 1.9% for males in 2015 (Fig. 1). The percentage curve for oral cancer out of all cancer types for males and females correlated with increases and decreases over the study period, apart from the years 1998 and 1999 (Fig. 1).

### ASR of oral cancer fluctuated over the study period

**Fig. 2:**
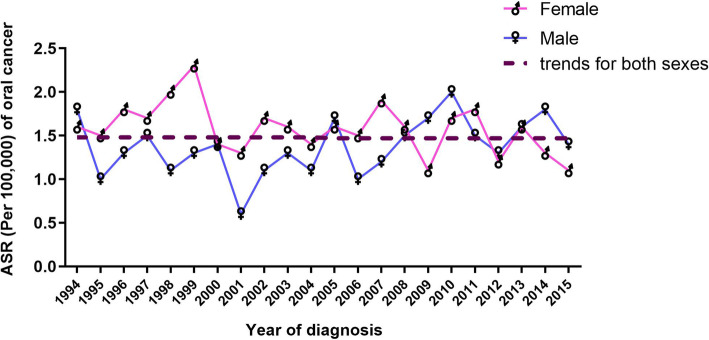
Age-standardised incidence rates (ASR) of oral cancer fluctuated over the study period. Between 1994 and 2015, the male ASR was 1.8 per 100,000 in 1994 and dropped to 0.6 in 2001. The female ASR fluctuated between 1.1 and 2.3 per 100,000

Between 1994 and 2015, the ASR per 100,000 male cases fluctuated: in 1994, it was 1.8, trending downwards to a low of 0.6 in 2001 and peaking at 2.0 in 2010 before dropping again to 1.4 in 2015 (Fig. 2). The female ASR per 100,000 increased from 1.6 in 1994 to a peak of 2.3 in 1999, decreasing again to 1.1 in 2015 (Fig. 2). For both sexes, ASR curves, like oral cancer percentages, correlate to increases and decreases over the study period (apart from the years 1998, 1999 and 2009) (Fig. 2) and generally remained constant from 1994 to 2015.

### AIR of oral cancer increases with age

**Fig. 3:**
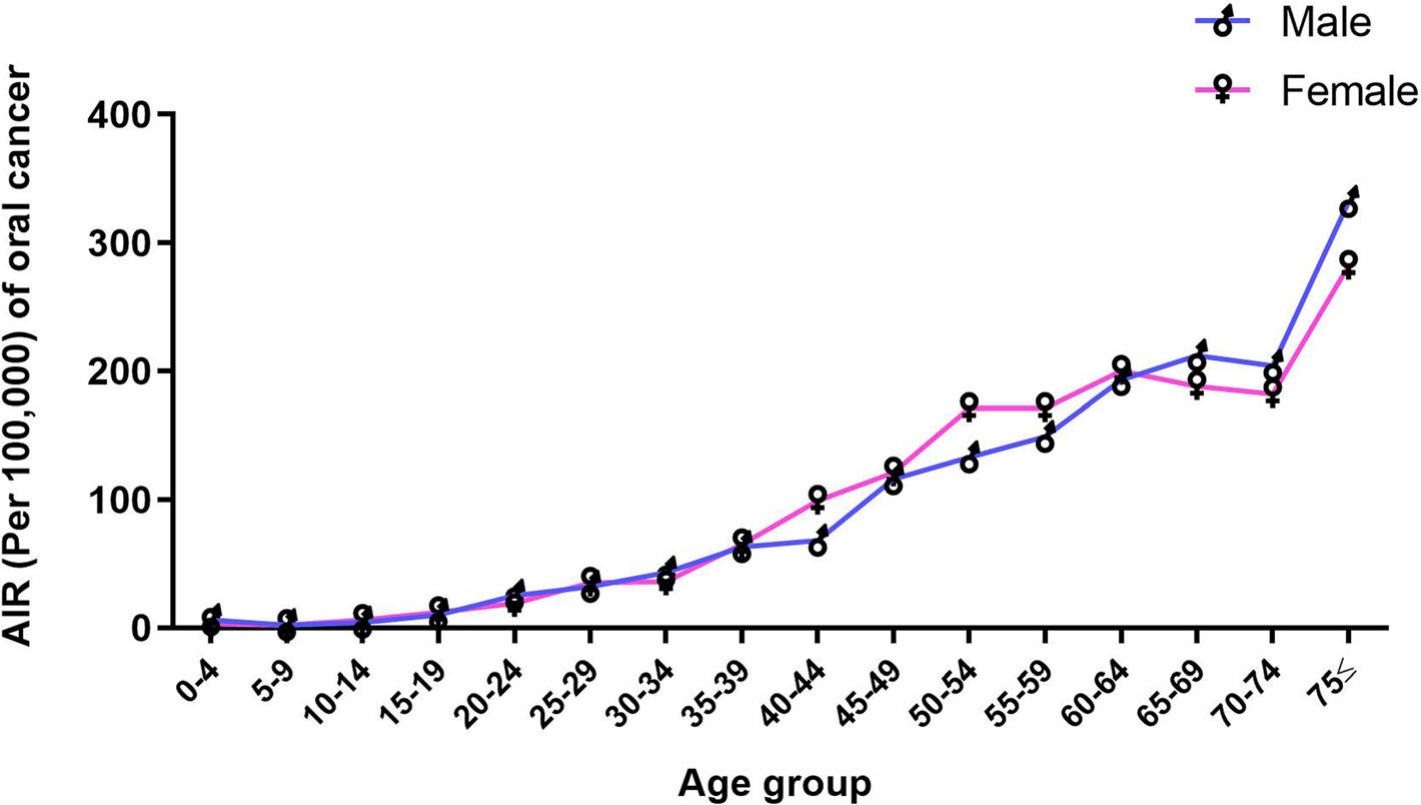
Age-specific incidence rates (AIR) of oral cancer increases with age. The total AIR of oral cancer increased noticeably with up until patients were 75 and over. More than 75% of the cases were diagnosed after the age of 50

The AIR data from 1994 to 2015 showed a positive correlation between oral cancer incidence and age, with most cancer cases occurring in the older age groups. Figure 3 shows the AIR of oral cancer increasing noticeably with age up until age 75. More than 75% of cases were diagnosed after the age of 50.

Some AIR differences were found between the sexes across age groups. From ages 35 to 64, rates of oral cancer were higher in females than in males; however, this trend had reversed to favour males in the 75-and-over age group. The overall AIR per 100,000 showed only slight differences between the sexes, at 33.6 for females and 31.2 for males (Fig. 3).

### ASR of oral cancer varies by region

**Fig. 4:**
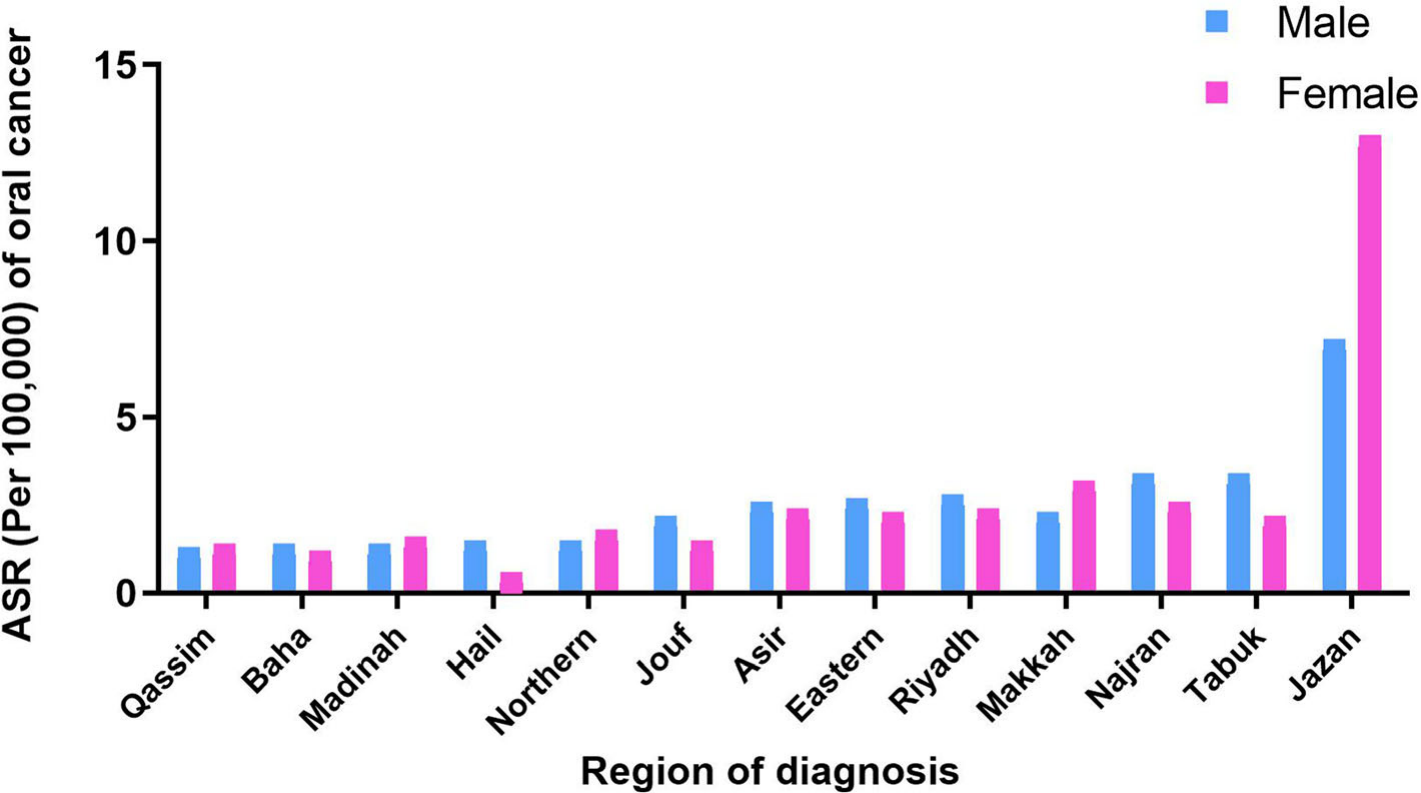
Age-standardised incidence rates (ASR) of oral cancer varies by region in Saudi Arabia. For all persons, the ASR means per 100,000 people for the period from 1994 to 2015 ranged from 1.5 in the Hail region to 19.6 in the Jazan region

The ASR data for oral cancer cases of all persons demonstrated a wide variation across Saudi regions. The ASR means per 100,000 people for the period from 1994 to 2015 ranged from 1.5 in Hail to 19.6 in Jazan, with a national average of 4.8 per 100,000 (Fig. 4).

The Jazan region had the highest male ASR mean at 6.9, followed by the Najran and Tabuk regions at 3.1 each (Fig. 4). Conversely, Qassim, Baha, Hail and the Northern province reported the lowest ASR averages at 1.0, 1.1, 1.1 and 1.2 per 100,000, respectively (Fig. 4).

Male and female ASR data were generally equivalent in terms of region rankings, with the Jazan region posting the highest overall ASR of 12.7 as an average value of both genders, followed by the Makkah region at 2.9 and the Najran region at 2.3 (Fig. 4). Similarly, the Hail, Baha, Qassim and Madinah regions posted the lowest ASR averages at 0.3, 0.9, 1.1 and 1.3, respectively (Fig. 4).

## Discussion

A review of oral cancer data in Saudi Arabia for the period from 1994 to 2015 showed an overall increasing trend in the numbers of oral cancer patients. Despite this rise, ASR data trends for oral cancer remained constant from 1994 to 2015 (Fig. 2). This curve stabilised in the face of a substantial Saudi Arabian population increase from 18.3 million in 1994 to 31.6 million in 2015 [9]. Many accumulative factors could be contributed to this stability. First, the significant increased access to health services in Saudi regions has contributed to the dissemination of oral health awareness and early diagnosis of some cases of metaplasia that were discovered before they could develop into cancerous tumours. Second, the increased level of public health in the Kingdom is usually linked to an increase in the economic level of the country, and individuals may have contributed to this constancy, as many infectious factors, such as viruses and fungi, have been linked to oral cancers. Third, Saudi Arabia is a majority Islamic country, wherein many oral cancer risk factors, such as alcohol consumption and cigarette smoking, are forbidden by Islamic law. Islamic law may thus mediate the lower number of oral cancer cases in Saudi Arabia compared to the rest of the world [10]. Thus, based on the IARC data for 2018, eight of the nine world regions whose ASR of oral cancer is above the global rate [5, 9] were located in non-Muslim countries [2], with Melanesian regions having the highest rate [2]. In contrast, most of the regions located within Muslim countries were ranked below the global ASR [2]. Further investigation of this aspect could therefore be valuable to cancer prevention efforts.

The ASR data revealed that more females than males were diagnosed with oral cancer in Saudi Arabia, at 1.4 for men and 1.6 for women. This finding is in contrast with global data showing that men are more likely to develop oral cancer than women [2]. In 2018, the most recent year for which IARC information is available, the global ASR of oral cancer was 5.2 for men and 2.3 for women [2]. While these rates do not match the global sex distribution, oral cancer in Saudi Arabia has a relatively low overall ASR when compared to the global average, as discussed above.

Results also revealed consistency in the ASR oral cancer curve for both sexes (Fig. 2), potentially due to the presence of common risk factors for oral cancer in males and females. This finding could be used as a starting threshold for studying the risk factors of oral cancer in the Saudi population through studying the common factors between the sexes.

As with many other types of cancer, the present study found a correlation between the occurrence of oral cancer and age, with 75% of cases diagnosed after the age of 50 years. In the USA, the average age at diagnosis of oral cancer is 62 years, and two-thirds of individuals with this disease are over the age of 55 [3]. Ageing is accompanied by increased susceptibility to cancer-causing genetic maturations due to accumulated exposures to environmental and behavioural risk factors. Avoiding these risk factors could greatly reduce the role that ageing plays in cancer.

This study found a wide variation in the incidence of oral cancers among Saudi regions. Such differences could indicate that regional environmental factors and lifestyle habits affect oral cancer incidence. The results reviewed above found that the Jazan region possessed the highest ASR of people with oral cancer. In contrast, the Northern province presented the lowest ASR. Several studies have focused on investigating why the Jazan region has such a high incidence of oral cancer [11–13]. Ibrahim et al. and others focused on the association of certain eating habits and lifestyle behaviours with the development of oral cancer, especially the abuse of *shamma*, a form of smokeless tobacco, and the chewing of *khat* (*Catha edulis*) leaves. These substances have been classified as carcinogens, especially in relation to oral cancer. Studies by these researchers found that consuming shamma increased the odds of developing oral cancer 29-fold, suggesting a strong link between oral cancer and diet and lifestyle choices.

According to the above, poor dietary habits related to tobacco use and its derivatives are one of the main reasons for the high incidence of oral cancer in some cities and not others. Other factors, such as variations in the genetic background of the Saudi regions’ citizens, cannot be excluded, especially because most of the population in the Kingdom’s regions is tribal, so consanguineous marriages are highly common. Thus, genomic sequencing can provide information on genetic variants that may be present in citizens of these regions and that may be linked with increased or decreased rates of oral cancer development. Population-based genetic testing is suggested.

## Conclusion

Despite the presence of year-to-year changes in the incidence of oral cancer in the Saudi population, there was overall no noticeable change in the incidence of oral cancer in the Saudi Arabian population for the period between 1994 and 2015. In contrast to some international findings, females were somewhat more likely than males to be diagnosed with oral cancer in Saudi Arabia. The positive correlation between ageing and the incidence of oral cancer for both males and females demonstrates that oral cancer is mainly a disease of the elderly, both in Saudi Arabia and across the globe. The wide variation in the incidence rates among Saudi regions raises an important research question concerning potential causes that need to be investigated further. The knowledge produced by this study must be translated into interventions by performing in-depth analyses of regional differences. This will contribute to the efforts of preventing oral cancer in Saudi Arabia.

## Data Availability

The data that support the findings of this study are available from Saudi Ministry of Health, but restrictions apply to the availability of these data, which were used under authorization for the current study.

## Abbreviations

AIR: Age-standardised incidence rates
ASR: Age-standardised specific rates
IARC: International Agency for Research on Cancer
SCR: Saudi Cancer Registry
ICD-10: International Classification of Diseases, 10th revision
